# Acute Generalized Exanthematous Pustulosis Induced by Fluconazole: A Case Report

**DOI:** 10.7759/cureus.106240

**Published:** 2026-03-31

**Authors:** Hajar El Hassani Taib, Meryem El Moustaoui, Najoua Ammar, Syrine Hamada, Nadia Ismaili, Laila Benzekri, Mariame Meziane

**Affiliations:** 1 Dermatology, Ibn Sina University Hospital Center, Mohammed V University, Rabat, MAR

**Keywords:** acute generalized exanthematous pustulosis (agep), fluconazole, hypersensitivity reaction, intertriginous areas, severe drug eruption

## Abstract

Acute generalized exanthematous pustulosis (AGEP) is a rare, severe type IV hypersensitivity reaction, most commonly triggered by antibiotics, calcium channel blockers, or antifungal agents, while fluconazole-induced AGEP is exceptionally reported. Early recognition and prompt management are essential to prevent complications. We report the case of a 21-year-old female with no personal or family history of psoriasis who developed a sudden-onset, pruritic, erythematous pustular rash six days after a single 150 mg dose of fluconazole prescribed for vaginal candidiasis. On examination, she was febrile (39°C) and asthenic, with widespread non-follicular pustules initially involving intertriginous areas and subsequently extending to the trunk and limbs. The Nikolsky sign was negative, and mucosal surfaces were spared. Laboratory investigations revealed leukocytosis (10,400/mm³) with neutrophilia (7,800/mm³), and pustular cultures were sterile. Histopathological examination showed intraepidermal pustules associated with dermal neutrophilic and eosinophilic infiltrates. The European Study of Severe Cutaneous Adverse Reactions (EuroSCAR) score was 11, confirming the diagnosis of fluconazole-induced AGEP. Rapid clinical improvement was observed following discontinuation of fluconazole and supportive treatment with antihistamines and emollients; desquamation occurred within four days, and no systemic complications were noted. Although rare, fluconazole should be considered a potential cause of AGEP, and early diagnosis with prompt withdrawal of the offending drug remains essential to ensure a favorable prognosis.

## Introduction

Acute generalized exanthematous pustulosis (AGEP) is a rare but severe type IV drug hypersensitivity reaction, most commonly triggered by antibiotics, calcium channel blockers, and other medications [1]. Fluconazole-induced AGEP is exceptionally rare. It presents with the sudden onset of numerous small, non-follicular, pruritic pustules on erythematous plaques, often affecting intertriginous and flexural areas [2,3]. Lesions usually resolve rapidly after discontinuation of the causative drug, and management involves supportive care with topical or, in severe cases, systemic corticosteroids [1].

We report here a case of fluconazole-induced AGEP in a young patient.

## Case presentation

A 21-year-old female patient with no personal or family history of psoriasis was hospitalized in our department for a sudden-onset erythematous, pruritic, and febrile rash occurring six days after a single 150 mg dose of fluconazole prescribed for vaginal candidiasis.

**Figure 1:**
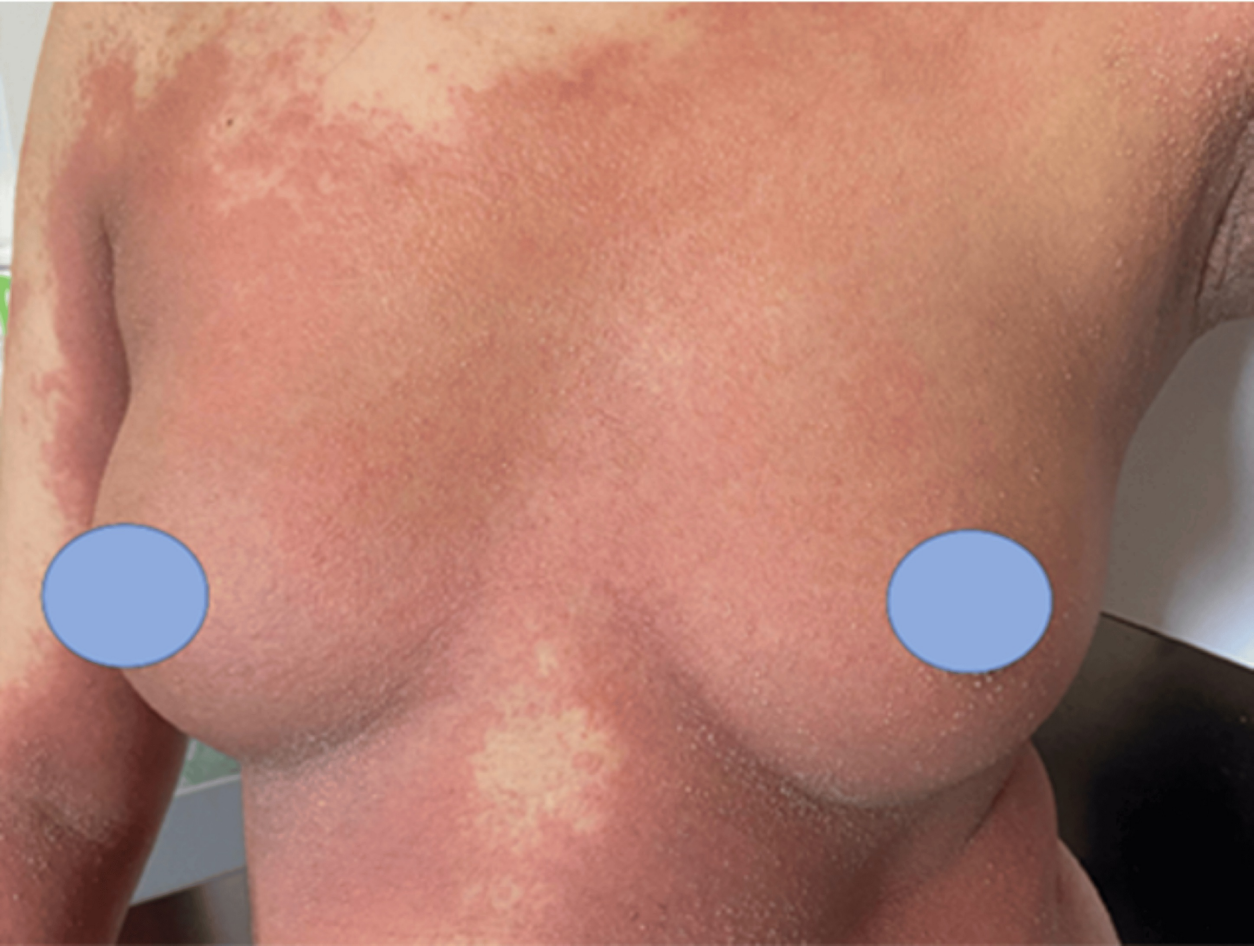
Maculopapular exanthem on the trunk, topped with multiple non-follicular pustules in the submammary folds

**Figure 2:**
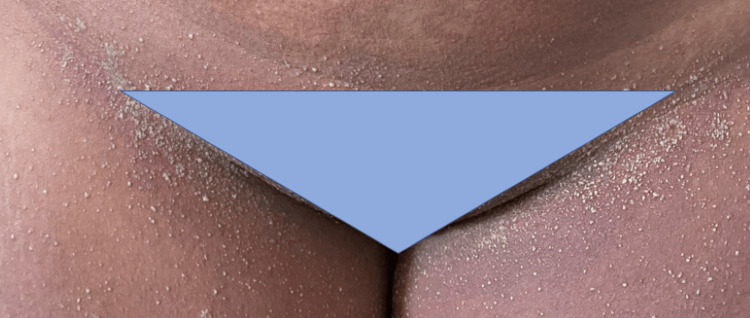
Exanthem topped with non-follicular pustules in the inguinal folds

**Figure 3:**
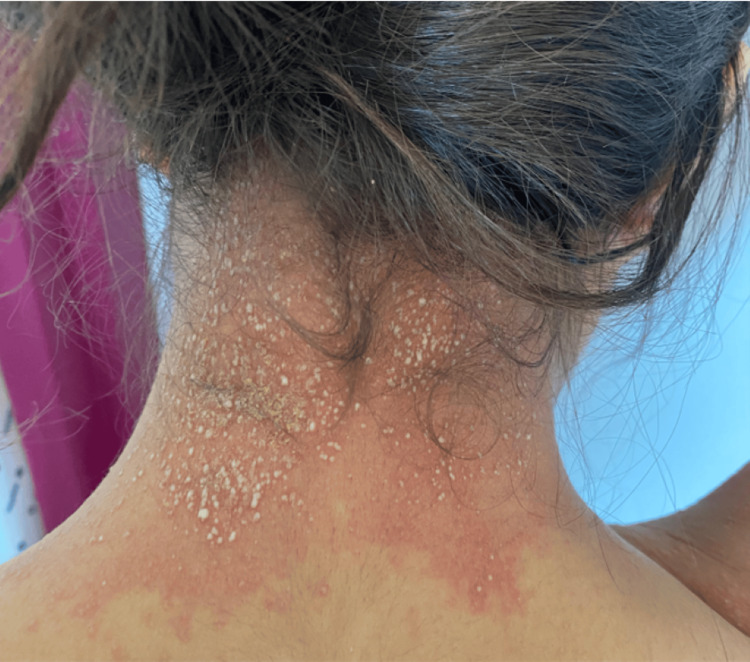
Exanthem topped with non-follicular pustules in the back of the neck.

Clinical examination revealed a febrile (39°C) and asthenic patient presenting with a pustular eruption on an erythematous background, initially involving the intertriginous areas and subsequently spreading to the rest of the body (Figures 1, 2, 3). The Nikolsky sign was negative, and mucosal examination was unremarkable. The patient reported no other recent drug intake.

Laboratory investigations showed leukocytosis (10,400/mm³) with neutrophilia (7,800/mm³), without hepatic or renal abnormalities. Microbiological examination of pustular swabs was sterile.

A skin biopsy was performed, and histological analysis demonstrated intraepidermal spongiform pustules associated with a dermal infiltrate of neutrophils and eosinophils.

In the present case, the diagnosis of AGEP induced by fluconazole was established based on clinical, biological, and histological findings, with a European Study of Severe Cutaneous Adverse Reactions (EuroSCAR) score of 11. Alternative diagnoses, including pustular psoriasis, toxic epidermal necrolysis, generalized exanthematous and pustular dermatophytid, and other cutaneous infections (particularly bacterial and viral), were considered unlikely given the acute onset, the clinical presentation, the absence of mucosal involvement, and the favorable outcome following discontinuation of fluconazole.

This case was reported to the pharmacovigilance unit, and causality was assessed using the French method, which identified fluconazole as the likely causative agent, with an intrinsic imputability score of I3 (probable), reflecting a plausible chronological and clinical relationship, and an extrinsic score of B4, indicating consistency with previously reported data in the literature.

The patient was treated with antihistamines and emollients, resulting in significant clinical improvement, with large-scale desquamation occurring four days after discontinuation of fluconazole. No systemic or topical corticosteroids were administered. The patient remained hospitalized for four days, and no additional supportive measures were required.

## Discussion

Acute generalized exanthematous pustulosis (AGEP) is a rare but potentially severe drug-induced eruption, most commonly associated with antibiotics, calcium channel blockers, and azole antifungals, including fluconazole, a widely prescribed triazole in dermatology. Despite its frequent use, fluconazole has been implicated in only a limited number of cases reported in the literature [2,4,5,6].

Several cases of fluconazole-induced AGEP have been described in both pediatric and adult patients aged between 7 and 70 years (Table 1) [2,4,5,6].

**Table 1:** Summary of published cases of fluconazole-induced AGEP and the present case. EuroSCAR: European Study of Severe Cutaneous Adverse Reactions; AGEP: acute generalized exanthematous pustulosis

Reference (year)	Fabre et al (2002) [6]	Di Lernia & Ricci (2015) [4]	Saliba et al. (2020) [5]	Chabbouh et al. (2024) [2]	This Case (2024)
Patient Age/Sex	65-year-old woman	70-year-old men	7-year-old boy	68-year-old woman	21-year-old woman
Indication for Fluconazole	Cutaneous candidosis on buttocks	Tongue candidiasis	Tinea capitis	Oral candidiasis	Vaginal candidiasis
Fluconazole Dosage	200 mg/day	150 mg once daily	Oral fluconazole (dose not specified )	Fluconazole (dose not specified )	150 mg single dose
Onset after Fluconazole	After the third dose	4 days after starting fluconazole (stopped after 2 days)	1 day prior to presentation	8 days after fluconazole intake	6 days after fluconazole intake
Clinical Presentation	Pustular eruption with erythema (trunk, large skin folds); fever (39°C); asthenia	Diffuse, rapidly progressing rash (trunk, limbs); fever (up to 38.5°C); malaise; numerous non-follicular, pinhead pustules (confluent in areas); purpuric lesions on limbs; erythema, edema, blisters on palms/soles	Diffuse, rapidly progressing rash (trunk, limbs, face); fever; multiple generalized non-follicular-based pinhead pustules on widespread erythema (confluent in areas)	Sudden onset erythematous and febrile eruption; multiple non-follicular, more or less coalescing pustules (predominantly on trunk and large folds); atypical cockades on palms; Nikolsky sign negative; no mucosal involvement	Sudden onset rash; fever; generalized pustular eruption on erythematous background; feeling unwell; Nikolsky sign negative; no mucosal involvement
Associated Symptoms	Fever, asthenia	Fever, malaise	Fever	Fever	Fever, general malaise
Laboratory Findings	Neutrophilia (9,000/mm³)	Increase in serum creatinine (1.9 mg/dl) and urea (29 mg/dl); WBC count 11,050 cells/mm³ with elevated neutrophils (80%); polycythemia; sterile pustule/blood cultures	Leukocytosis with neutrophilia; sterile scalp swab culture (Microsporum gypseum identified as underlying infection)	Hyperleukocytosis with neutrophilia predominance; normal hepatic and renal function; negative bacteriological and mycological cultures of pustules	Leukocytosis (10,400/mm³) with neutrophilia (7,800/mm³); sterile pustule culture
Histology	Consistent with AGEP	Subcorneal pustules within stratum corneum; papillary dermis edema; mixed superficial and perivascular inflammatory infiltrate (neutrophils, lymphocytes, few eosinophils)	Spongiform subcorneal and intraepidermal pustules; mixed superficial and perivascular inflammatory cells (neutrophils, lymphocytes, eosinophils); necrotic keratinocytes in epidermis	Multilocular intra-epidermal pustules; dermal infiltrate of neutrophils and eosinophils; no acanthosis or parakeratosis	Intra-epidermal pustules; dermal infiltrate of neutrophils and eosinophils
Diagnosis Confirmed By	Diagnosis based on clinical, laboratory, and histological findings; EuroSCAR score classified as certain	Clinical and histological findings; EuroSCAR score 12 (definite AGEP)	Clinical presentation; punch biopsy	Clinical presentation; onset time; histological and evolutionary data; EuroSCAR score 9 (definite AGEP)	Clinical presentation; histology; EuroSCAR score 11 (definite AGEP); French imputability I3, B4
Treatment	Local steroids	Methylprednisolone (systemic) + topical steroids (betamethasone dipropionate 0.05% cream)	Topical steroid (clobetasol propionate); oral Griseofulvin (for tinea capitis)	Topical corticosteroids	Topical steroids; antihistamines; emollients
Outcome	Eruption cleared in about ten days; recurrence 19 days later (without fever/neutrophilia)	Eruption quickly improved, pustulation ceased within a few days; widespread desquamation followed	Resolution of rash after 4 weeks; full hair regrowth at 4 weeks follow-up	Marked improvement and large-scale desquamation within 5 days after fluconazole discontinuation	Significant improvement and large-scale desquamation within 4 days after fluconazole discontinuation
Patch Test	Not mentioned in source	Positive fluconazole patch test after 48h	Not mentioned in source	Not mentioned in source	Not performed
Rarity/Significance	First reported case	"Single case report" prior to this one, making it the second reported	Fourth reported case	Fluconazole "exceptionally described in the literature"; new case report	Adds to the very limited number of reported cases of fluconazole-induced AGEP

These cases typically presented with a sudden onset of widespread erythematous eruptions covered by numerous non-follicular pustules, initially involving intertriginous or truncal areas before rapidly spreading to the limbs. Fever, asthenia, and neutrophilia were common, while mucosal involvement was consistently absent [2,4,5,6]. Systemic manifestations were uncommon, with only one reported case showing transient renal dysfunction [4]. Histopathological findings were consistent across cases, demonstrating intraepidermal or subcorneal pustules associated with a neutrophil-rich dermal infiltrate, and negative microbiological cultures [2,4,5,6]. Resolution generally occurred within days to weeks following discontinuation of fluconazole, with or without supportive treatment, although recurrence after nineteen days was reported in one case [6]. 

Compared with previously reported cases, our observation is consistent in terms of clinical presentation and biological findings. Notably, our patient showed rapid clinical improvement without the use of corticosteroids, with complete resolution occurring within four days after drug discontinuation. This highlights the self-limited course of fluconazole-induced AGEP and further supports the causal role of the drug.

Acute generalized exanthematous pustulosis is characterized by an acute onset, typically within 24-48 hours after exposure to the causative drug, although delays of up to several days may occur depending on the medication [7]. It presents with fever (>38°C), asthenia, and a rapidly spreading erythematous rash followed by the appearance of numerous sterile, non-follicular pustules, predominantly affecting the trunk and intertriginous areas, and usually sparing the mucous membranes. Mucosal involvement is reported in approximately 20% of cases and is generally limited to a single site [8]. Systemic involvement has been described in up to 17% of cases, affecting organs such as the liver, kidneys, bone marrow, and lungs [9]. Histologically, AGEP is characterized by intraepidermal or subcorneal pustules, papillary dermal edema, and a perivascular infiltrate composed mainly of neutrophils and eosinophils, with possible spongiform changes and keratinocyte necrosis [7].

The diagnosis relies on a combination of clinical, biological, and histological findings, supported by the EuroSCAR scoring system [10]. In the present case, the diagnosis of fluconazole-induced AGEP was established based on the temporal relationship with drug intake, clinical presentation, laboratory and histological findings, and disease course. The differential diagnosis includes infectious conditions (such as cutaneous candidiasis, bacterial folliculitis, and herpes simplex virus infection), inflammatory dermatoses (including pustular psoriasis, immunoglobulin A pemphigus, and subcorneal pustular dermatosis), and severe drug reactions such as Drug Reaction with Eosinophilia and Systemic Symptoms (DRESS), Stevens-Johnson syndrome, and toxic epidermal necrolysis. These conditions can be excluded based on clinical features, histological findings, and appropriate microbiological investigations, including pustule cultures and, when indicated, polymerase chain reaction (PCR) testing for viral pathogens [3].

The cornerstone of treatment is the immediate discontinuation of the causative drug. Management is mainly supportive, including emollients, antihistamines, and topical corticosteroids, while systemic corticosteroids may be considered in more severe cases [8,11]. Alternative therapies such as cyclosporine, secukinumab, or infliximab have been reported in refractory cases [12,13,14].

Fluconazole-induced AGEP is classified as a type IVd delayed hypersensitivity reaction mediated by drug-specific T lymphocytes [5]. Several mechanisms have been proposed, including the hapten/prohapten model, the pharmacological interaction (p-i concept), and the altered peptide repertoire model [4]. These mechanisms lead to activation of CD4+ and CD8+ T cells, which release neutrophil-attracting cytokines such as interleukin-8, resulting in sterile pustule formation [4,5]. Positive patch tests reported in some cases further support an immunologically mediated mechanism [5].

## Conclusions

This case highlights the importance of early recognition and timely management of acute generalized exanthematous pustulosis (AGEP) induced by fluconazole. AGEP is a rare but serious drug reaction that requires prompt discontinuation of the causative medication and supportive care. The clinical presentation, along with histological findings, is key to the diagnosis. With appropriate treatment, the prognosis is generally favorable, emphasizing the need for vigilant monitoring of patients on high-risk medications.

Fluconazole is widely prescribed in dermatology, and clinicians should consider it as a potential inducer of AGEP. However, as this is a single case report, these conclusions should be interpreted with caution and may not be generalizable without further studies.
